# Mpox, stigma and the Public Health Emergency of Continental Security declaration: Addressing public health challenges in Africa

**DOI:** 10.4102/jphia.v15i1.757

**Published:** 2024-08-30

**Authors:** Morẹ́nikẹ́ Oluwátóyìn Foláyan

**Affiliations:** 1 Department of Child Dental Health, Obafemi Awolowo University, Ile-Ife, Nigeria; 2 Oral Health Initiative, Centre for Population Studies, Nigeria Institute of Medical Research, Yaba, Lagos, Nigeria

On 12 August 2024, the Africa Centre for Disease Control and Prevention (CDC) declared mpox outbreaks a Public Health Emergency of Continental Security (PHECS) in Africa. The decision was driven by the escalating mpox situation in Africa, where since 2022, 40 874 cases (1512 deaths) have been reported across 17 African Union member states.^1^ In 2024, the continent saw a 160% increase in cases and a 19% increase in deaths compared to the same period in 2023.^1^ Between January 2024 and 20 August 2024, 18 910 cases (3154 confirmed; 15 756 suspected) and 541 deaths (case fatality rate [CFR]: 2.95%) were reported with no cases reported from North Africa.^1^

Children under 15 years account for 60% of reported cases and the population report a high CFR (> 3.9%). Children under the age of one are four times more likely to die compared to those over the age of 15 years.^2^ Mpox is now transmitted by heterosexual contact in Africa, irrespective of the clade type.^3,4^ This presents a different profile from the high transmission rate among men who have sex with men in Europe.^5^ The prevalence of the mpox is also higher among women (53.8%), including female sex workers^4^ indicating that heterosexual transmission may be driving the outbreak in Africa.

Vulnerable populations, particularly those living with human immunodeficiency viruses (HIV) and pregnant women, face heightened risks of severe mpox because of compromised immune systems.^6^ This raises concerns about vertical transmission risks and adverse pregnancy outcomes.^7,8^ Despite the increasing number of cases, the response has been hampered by a low detection rate – as low as 39% – and inadequate resources, resulting in a severe underestimation of the outbreak’s true scale. Compounding the challenge is cross-border movements of people that have spread the virus to new regions, including urban areas with limited response experience.

The PHECS declaration emphasises the need for strategic action and response to control HIV in Africa, with a particular focus on community response. This critical aspect can significantly influence the success or delay of outbreak control. Lessons learned from the HIV pandemic, Ebola epidemic and coronavirus disease 2019 (COVID-19) pandemic highlight the importance of planned and strategic interventions through active community engagement. However, these efforts are often hindered by stigmatisation and the accompanying discrimination.

Stigma surrounding mpox can significantly undermine public health efforts. Much like the early days of HIV and/or acquired immunodeficiency syndrome (AIDS), individuals infected or suspected of having mpox may experience social ostracisation, discrimination and even violence. This stigma not only discourages people from seeking medical care but also leads to underreporting and delayed diagnoses, thereby hindering containment efforts. In African societies, where cultural and social norms are vital to community health, addressing stigma is crucial for effective disease management. If strategic interventions are not prioritised as a critical first-line of response, a PHECS declaration could exacerbate the risk of stigma resulting from infodemic and conspiracy theories regarding infection emergence, transmission and spread.^9^

During the public health experts’ meeting that led to the PHECS declaration, there was a consensus on the urgency of the situation and the lack of comprehensive data. Stigma associated with the disease was identified as one of the 18 criteria for making the PHECS declaration. The public response must therefore address both the epidemiological aspects of mpox and its social dimensions, including stigma and discrimination. Many of the stigmatising attitudes and behaviours towards those with mpox are likely to mirror those seen with HIV and/or AIDS, Ebola and COVID-19 diseases that are transmissible, life-threatening, and without a medical cure. Similar to HIV, mpox disproportionately affects historically criminalised and marginalised populations, such as female sex workers in Africa, to the non-exclusion of men who have sex with men, because of the high sex network. In addition, people living with HIV face a higher risk of severe mpox infection, with a risk of compounding the stigma the community already experience. Unlike HIV and/or AIDS, however, mortality from mpox occurs over a shorter time span, although not as rapidly as with Ebola or COVID-19.

To effectively combat stigma, Africa CDC as the coordinator of the mpox response in Africa, and its country and community partners, must quickly launch public health campaigns aimed at reducing the stigma associated with the disease. These campaigns should prioritise education, community engagement, and support for affected individuals, encouraging early reporting, testing and treatment. Public messaging should clearly state that mpox spreads through physical contact or contact with body fluids, not through sexual orientation, and should avoid reinforcing stereotypes. Poor risk communication whether through interpersonal interactions, media, or healthcare settings can exacerbate stigma.^10^ Anonymised testing and contact tracing should encourage individuals to seek medical care promptly.

Enhancing surveillance and data collection efforts will help accurately assess the extent of the outbreak and guide decisions on deploying personnel for community engagement programmes in each country. This is an opportune moment for Africa CDC to strategically implement its community health workers engagement plan. In addition, mpox resource allocation should prioritise building the capacity of community health workers for disease prevention, detection and appropriate referral.

It is important that the response to the PHECS declaration must be an integrated unified response to addressing both its medical and social dimensions, such as stigma and discrimination.^2^ A research agenda focused on the epidemiology of mpox in Africa, vaccination strategy effectiveness, and the impact of stigma is crucial for developing successful interventions. The decision-making process should consciously ensure the active engagement of community actors as these voices are not only essential but also critical at this formative stage of the mpox response in Africa. Meanwhile, insights gained from managing stigma and discrimination in previous infectious diseases such as HIV and/or AIDS, Ebola and COVID-19 can guide the design and implementation of strategies to mitigate these issues during the current mpox outbreak in Africa.

In conclusion, mpox poses a significant public health challenge in Africa and the threat can be exacerbated by the stigma surrounding the disease. Mitigating stigma and discrimination through a coordinated and multifaceted first-line response would be strategic for timely control of the outbreak in Africa. The Africa CDC’s leadership, support of the international community, and active engagement of civil society organisation, community advocates and community health workers will be crucial in ensuring an effective mitigation of the impact of stigma and discrimination that may result from the Public Health Emergency of Continental Security declaration.

## References

[1] Africa CDC. Africa CDC Epidemic Intelligence Weekly Report, August 2024 [homepage on the Internet]. [cited 2024 Aug 15]. Available from: https://africacdc.org/download/africa-cdc-weekly-event-based-surveillance-report-august-2024/

[2] Tomori O. Neglected mpox resurges with virulence in Africa: Will this be another neglected warning? J Public Health Afr. 2024;15(1):a730. 10.4102/jphia.v15i1.730 PMC11369574 39229340

[3] Masirika LM, David F, Nieuwenhuijse DF, et al. Mapping the distribution and describing the first cases from an ongoing outbreak of a New Strain of mpox in South Kivu, Eastern Democratic Republic of Congo between September 2023 to April 2024. medRxiv [preprint]; 2024. 10.1101/2024.05.10.24307057

[4] Katoto PD, Muttamba W, Bahizire E, et al. Shifting transmission patterns of human mpox in South Kivu, DR Congo. Lancet Infect Dis. 2024;24(6):e354–e355. 10.1016/S1473-3099(24)00287-1 38703785

[5] Vries HJ, Götz HM, Bruisten S, et al. Mpox outbreak among men who have sex with men in Amsterdam and Rotterdam, the Netherlands: No evidence for undetected transmission prior to May 2022, a retrospective study. Euro Surveill. 2023;28(17):2200869. 10.2807/1560-7917.ES.2023.28.17.2200869 37103788 PMC10283470

[6] Ahmed SK, Mohamed MG, Dabou EA, et al. Monkeypox (mpox) in immunosuppressed patients. F1000Res. 2023;12:127. 10.12688/f1000research.130272.2 37089133 PMC10113800

[7] Velázquez-Cervantes MA, Ulloa-Aguilar JM, León-Juárez M. Mpox and pregnancy: A neglected disease and its impact on perinatal health. Rev Clin Esp (Barc). 2023;223(1):32–39. 10.1016/j.rceng.2022.09.002 36341988 PMC9620439

[8] Dashraath P, Nielsen-Saines K, Rimoin A, Mattar CNZ, Panchaud A, Baud D. Monkeypox in pregnancy: Virology, clinical presentation, and obstetric management. Am J Obstet Gynecol. 2022;227(6):849.e7–861.e7. 10.1016/j.ajog.2022.08.017 35985514 PMC9534101

[9] Ennab F, Nawaz FA, Narain K, et al. Monkeypox outbreaks in 2022: Battling another ‘pandemic’ of misinformation. Int J Public Health. 2022;67:1605149. 35910429 10.3389/ijph.2022.1605149 PMC9329665

[10] Banjar WM, Alaqeel MK. Monkeypox stigma and risk communication; Understanding the dilemma. J Infect Public Health. 2024;17 Suppl 1:4–7. 10.1016/j.jiph.2023.03.002 37002063 PMC10014508

